# Differing Efficacies of Lead Group A Streptococcal Vaccine Candidates and Full-Length M Protein in Cutaneous and Invasive Disease Models

**DOI:** 10.1128/mBio.00618-16

**Published:** 2016-06-14

**Authors:** Tania Rivera-Hernandez, Manisha Pandey, Anna Henningham, Jason Cole, Biswa Choudhury, Amanda J. Cork, Christine M. Gillen, Khairunnisa Abdul Ghaffar, Nicholas P. West, Guido Silvestri, Michael F. Good, Peter M. Moyle, Istvan Toth, Victor Nizet, Michael R. Batzloff, Mark J. Walker

**Affiliations:** a Australian Infectious Diseases Research Centre and School of Chemistry and Molecular Biosciences, The University of Queensland, St. Lucia, Queensland, Australia; b Institute for Glycomics, Griffith University, Gold Coast Campus, Southport, Queensland, Australia; c University of California, San Diego, San Diego, California, USA; d Emory Vaccine Center and Yerkes National Primate Research Center, Emory University, Atlanta, Georgia, USA; e School of Pharmacy, The University of Queensland, St. Lucia, Queensland, Australia

## Abstract

Group A *Streptococcus* (GAS) is an important human pathogen responsible for both superficial infections and invasive diseases. Autoimmune sequelae may occur upon repeated infection. For this reason, development of a vaccine against GAS represents a major challenge, since certain GAS components may trigger autoimmunity. We formulated three combination vaccines containing the following: (i) streptolysin O (SLO), interleukin 8 (IL-8) protease (*Streptococcus pyogenes* cell envelope proteinase [SpyCEP]), group A streptococcal C5a peptidase (SCPA), arginine deiminase (ADI), and trigger factor (TF); (ii) the conserved M-protein-derived J8 peptide conjugated to ADI; and (iii) group A carbohydrate lacking the *N*-acetylglucosamine side chain conjugated to ADI. We compared these combination vaccines to a “gold standard” for immunogenicity, full-length M1 protein. Vaccines were adjuvanted with alum, and mice were immunized on days 0, 21, and 28. On day 42, mice were challenged via cutaneous or subcutaneous routes. High-titer antigen-specific antibody responses with bactericidal activity were detected in mouse serum samples for all vaccine candidates. In comparison with sham-immunized mice, all vaccines afforded protection against cutaneous challenge. However, only full-length M1 protein provided protection in the subcutaneous invasive disease model.

## IMPORTANCE

This set of experiments demonstrates the inherent variability of mouse models for the characterization of GAS vaccine candidate protective efficacy. Such variability poses an important challenge for GAS vaccine development, as advancement of candidates to human clinical trials requires strong evidence of efficacy. This study highlights the need for an open discussion within the field regarding standardization of animal models for GAS vaccine development.

## INTRODUCTION

Group A *Streptococcus* (GAS) causes numerous human disease manifestations from mild infections of the skin and throat to more serious and life-threatening conditions, such as necrotizing fasciitis or toxic shock syndrome, to poststreptococcal autoimmune complications, including rheumatic heart disease and poststreptococcal glomerulonephritis. Globally, more than 500,000 deaths per annum are estimated to be caused by GAS-related diseases (1), and treatment of GAS infections poses a significant economic burden (2, 3). Though an efficacious vaccine against GAS would represent the best and most cost-effective intervention to diminish disease burden, the link between GAS infection and autoimmune sequelae has hindered the development of such a vaccine. More than 30 years ago, a ban on the administration of GAS and its components into humans was enforced by the U.S. Food and Drug Administration (FDA), after children vaccinated with GAS M protein developed rheumatic fever (4). Although this ban was subsequently lifted in 2005 (5), the quest to develop an effective and safe GAS vaccine for human use is ongoing.

A recent report by the World Health Organization (WHO) lists 26 vaccine candidates that have shown promising results in preclinical studies (6). While precluded as a human vaccine candidate due to safety concerns, full-length M protein from the homologous GAS challenge strain is effective at preventing infection in a variety of animal models and is regularly used as a positive control for GAS vaccine studies (7–9). However, the lack of a standardized animal model for GAS immunization studies has prompted the use of a diverse range of mouse models for evaluation of vaccine efficacy. A few of the many variations that have been reported for GAS vaccine assessment (10, 11) include the following: infections via the intraperitoneal, cutaneous, subcutaneous, intranasal, and intramuscular routes; measurement of survival, bacterial colonization, and dissemination; evaluation of passive and active protection; and differing adjuvant formulations. This diversity in models of protection used to assess vaccine efficacy has made it difficult to compare GAS vaccine compositions.

In this study, we selected promising vaccine candidates described previously to confer protection in at least one mouse model to formulate three experimental vaccines. Homologous M1 protein was used as a positive control, and phosphate-buffered saline (PBS) was used as a negative control. All vaccine formulations were adjuvanted with aluminum hydroxide (alum). The first experimental vaccine consisted of a combination of trigger factor (TF) (7) and inactivated versions of arginine deiminase (ADI) (7, 12), streptolysin O (SLO) (8, 13), *Streptococcus pyogenes* cell envelope proteinase (SpyCEP) (8, 14), and group A streptococcal C5a peptidase (SCPA) (15). The second experimental vaccine consisted of the conserved M-protein-derived J8 peptide (16, 17) conjugated to ADI, while the third vaccine contained group A carbohydrate lacking the *N*-acetylglucosamine side chain (ΔGAC) (18) conjugated to ADI. We evaluated the immunogenicity and efficacy of vaccine candidates in mice using a superficial skin infection model and invasive disease model upon challenge with M1 GAS, allowing parallel comparison between the different experimental vaccine formulations.

## RESULTS

### Antibody response to experimental GAS vaccine antigens.

**FIG 1:**
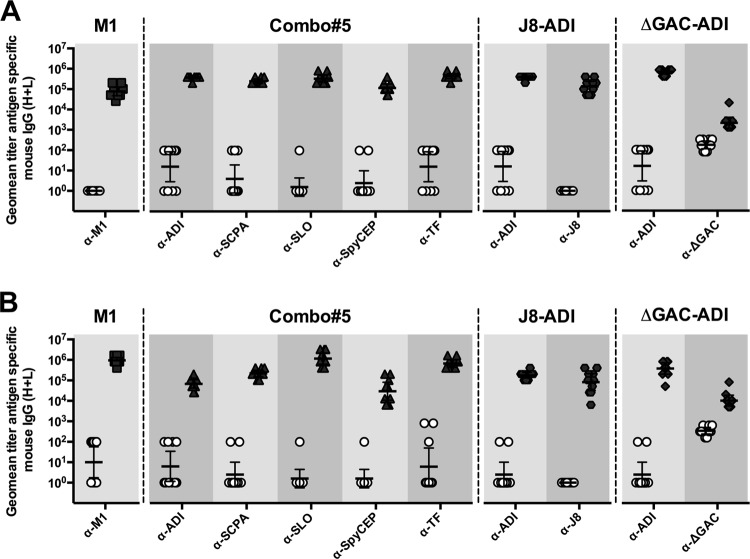
Antigen-specific IgG response in serum samples from BALB/c (A) and humanized plasminogen AlbPLG1 (B) mice at day 35 (*n* = 10). Antigens used to coat ELISA plates are displayed. IgG titers in antigen-immunized groups (M1, Combo#5, J8-ADI, or ΔGAC-ADI) were compared to titers in PBS-immunized mice (open circles) and found to be significantly different (*P* < 0.0001) using a two-tailed Mann-Whitney U test. Each symbol represents the value for an individual mouse. The bars show the geometric mean titer (geomean titer) with 95% confidence interval (95% CI). α-M1, anti-M1 antibody.

The immune response to each vaccine component was assessed by enzyme-linked immunosorbent assay (ELISA) following immunization with M1 protein, the five-component SLO, SpyCEP, SCPA, ADI, and TF vaccine (hereafter designated Combo#5), and the conjugate J8-ADI and ΔGAC-ADI vaccines. Both BALB/c and humanized plasminogen mice responses to all protein antigens (M1, ADI, TF, SLO, SpyCEP, and SCPA) were significantly higher in vaccinated mice than in sham-immunized control mice (Fig. 1). J8 peptide was successfully conjugated to ADI, with an average peptide-to-protein ratio of three peptide molecules per ADI molecule as determined by amino acid analysis. Immunization with this conjugate yielded both J8-specific and ADI-specific antibodies in sera of both mouse strains and with specific titers significantly higher in J8-ADI-vaccinated mice than in control PBS-immunized mice (Fig. 1). ΔGAC was also successfully conjugated to ADI, with an average glycan-to-protein ratio of 0.78 glycan molecule per ADI molecule as determined by quantification of protein and carbohydrate concentrations in the conjugate. Immunization with ΔGAC-ADI conjugate resulted in the generation of ΔGAC-specific and ADI-specific murine antibody titers significantly higher than those detected in PBS-immunized mice (Fig. 1).

**FIG 2:**
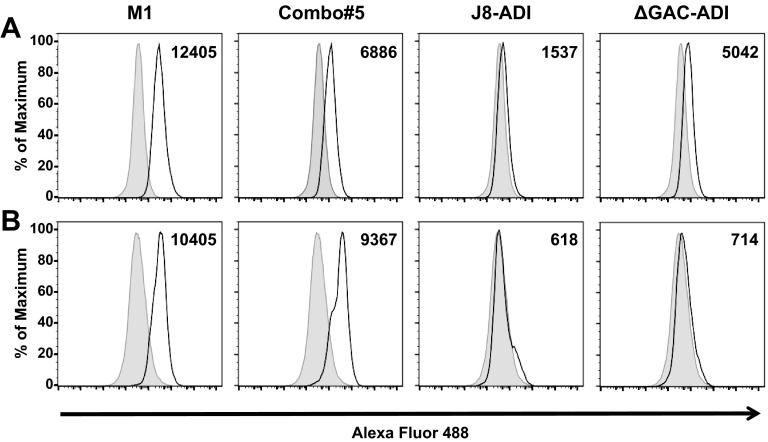
Antibody binding to the surfaces of GAS. Sera from immunized BALB/c (A) and humanized plasminogen *Alb*PLG1 (B) mice raised against vaccine antigens (M1, Combo#5, J8-ADI, and ΔGAC-ADI) were incubated with live GAS pM1.200 and 5448, respectively. Specific binding to the bacterial surface was detected by a shift in Alexa Fluor 488 fluorescence. Representative histograms for test antigens are shown with a black solid line; histograms for PBS-immunized mice are shown as a shaded area. The populations in histograms for test antigens were statistically different (*P* < 0.01) from control histograms with *T*(*X*) values above the established cutoff value of *T*(*X*) = 100. *T*(*X*) values, shown on the right top corner of each panel, were determined by the probability binning algorithm in FlowJo.

Additionally, we assessed the binding of murine serum antibodies to the surfaces of live GAS, as detected by flow cytometry. Incubation of serum samples from BALB/c mice with GAS pM1.200 showed a significant shift in fluorescence in sera from vaccinated groups (solid line) compared to sera from PBS-immunized mice (shaded histogram). Serum samples from M1- and Combo#5-immunized mice showed the largest shifts in fluorescence (Fig. 2A). The sera from antigen-vaccinated humanized plasminogen mice incubated with GAS 5448 also showed a significant shift in fluorescence (solid line) compared to sera from PBS-immunized mice (shaded histogram) (Fig. 2B).

### Bacterial opsonization.

**FIG 3:**
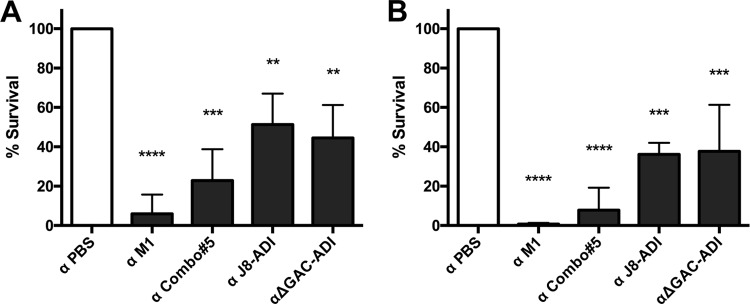
Indirect bactericidal assay. Pooled heat-inactivated sera raised against vaccine antigens in BALB/c (A) and humanized plasminogen *Alb*PLG1 (B) mice were preincubated with GAS pM1.200 and 5448, respectively. Human blood tested to support the growth of both strains was then added, and following 3-h incubation at 37°C, samples were plated for CFU determination. The survival percentage was calculated using the growth of GAS in serum from PBS-immunized mice as 100%. Values are the mean survival percentage plus standard deviation (SD) (error bars) for three independent replicates. Survival percentages were compared using one-way ANOVA corrected for multiple comparisons using Dunnett’s test. Values that are significantly different from the value for the control (α PBS) are indicated by asterisks as follows: **, *P* < 0.01; ***, *P* < 0.001; ****, *P* < 0.0001.

An indirect bactericidal assay was used to investigate the ability of heat-inactivated murine serum from vaccinated mice to enhance the killing of GAS in the presence of human blood. Serum from BALB/c mice was used to test bactericidal activity against GAS pM1.200 (Fig. 3A), while serum from humanized plasminogen mice was used with GAS 5448 (Fig. 3B). Our results are similar for both groups, with anti-M1 sera being the most opsonic, followed by anti-Combo#5 sera. Overall, all experimental vaccines were able to raise bactericidal antibodies compared to sera from sham-immunized mice.

### Murine model of skin infection.

**FIG 4:**
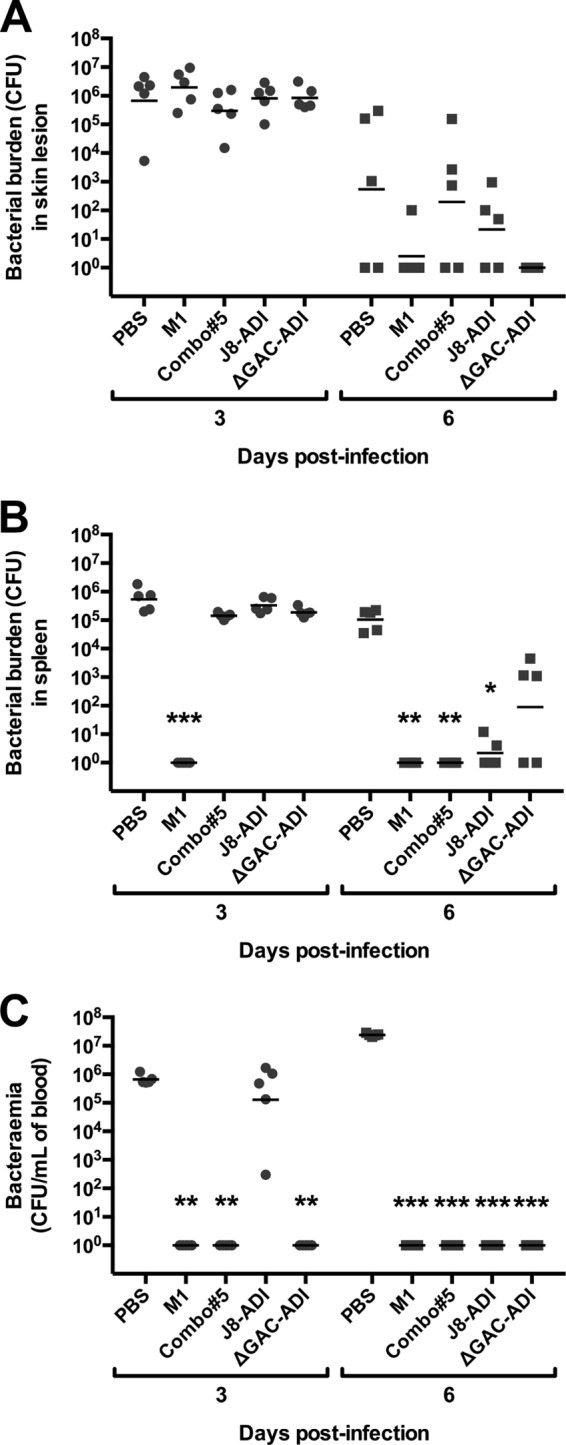
Bacterial persistence in the skin (A) and dissemination into spleen (B) and blood (C) following skin challenge of BALB/c mice (*n* = 10 for all groups). Mice were infected on day 42, and on days 3 and 6 postinfection, five mice per group were sacrificed to determine GAS bacterial burden. Each symbol represents the value for an individual mouse. The short lines represent geometric means of CFU. Statistical significance was determined by Kruskal-Wallis test corrected for multiple comparisons using Dunn’s test (*, *P* < 0.05; **, *P* < 0.01; ***, *P* < 0.001). Samples where GAS was undetected were allocated a value of 1 for graphical representation.

Protection against superficial skin infection with GAS pM1.200 in immunized BALB/c mice was determined by measuring bacterial persistence in skin lesions and dissemination of GAS into the blood and spleen. Five mice per group were euthanized on days 3 and 6 postinfection, and bacterial CFU were enumerated. In skin samples, a reduction in CFU was observed in samples from vaccinated mice compared to PBS-immunized mice on day 6 postinfection; however, this difference did not reach statistical significance (Fig. 4A). GAS pM1.200 was not detected in spleen samples from M1-immunized mice from day 3 postinfection onwards, while on day 6 postinfection, Combo#5- and J8-ADI immunized mice showed significantly lower CFU in the spleen compared to PBS-immunized mice (Fig. 4B). All vaccine antigens showed significant protection against bacteremia on day 6 postinfection compared to the PBS control. Furthermore, no detectable bacteria were found in samples from M1-, Combo#5-, and ΔGAC-ADI-vaccinated mice on day 3 postinfection (Fig. 4C).

### Murine model of invasive disease.

**FIG 5:**
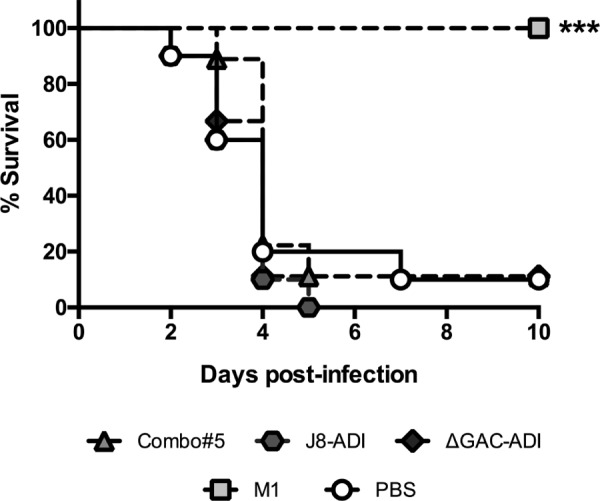
Survival of humanized plasminogen *Alb*PLG1 mice following subcutaneous challenge with 1.3 × 10^8^ CFU of GAS 5448 on day 42 (*n* = 10 for the PBS and J8-ADI groups; *n* = 9 for the Combo#5 and ΔGAC-ADI groups; *n* = 8 for the M1 group). Curves were compared using the log rank (Mantel-Cox) test (***, *P* < 0.001).

To assess vaccine efficacy against more severe forms of infection, an invasive mouse model of disease was employed. Following subcutaneous challenge of humanized plasminogen mice with GAS strain 5448, survival was monitored for 10 days. M1 protein was the only experimental vaccine that conferred protection against GAS strain 5448 challenge, with 100% survival (Fig. 5). Immunization with Combo#5, J8-ADI, and ΔGAC-ADI did not confer protection against lethal challenge beyond that observed in sham-immunized mice.

## DISCUSSION

In recent years, the emergence of a worldwide epidemic of invasive infections (19, 20), scarlet fever outbreaks (21, 22), and antibiotic-resistant GAS strains (23) has emphasized the urgent need for a safe and efficacious GAS vaccine for human use. A reduction in GAS carriage and superficial infection through an effective vaccine strategy would likely reduce the burden of serious GAS disease, with the additional benefit of reducing antibiotic prescription and potentially leading to reduced levels of antibiotic resistance. GAS vaccine candidates can be classified into two categories, M-protein-based vaccines and non-M-protein-based vaccines. M-protein-based vaccines target either the hypervariable or conserved domain of the M protein. Candidate vaccines targeting the hypervariable region of M protein follow a multivalent vaccine approach, where the hypervariable domain of M protein from selected serotypes are included in the formulation (24–26). Most recently, one such multivalent vaccine was formulated to include 30 serotypes (26). While cross-opsonization has been observed against some serotypes not included in the formulation (26), heterologous protection in animal models has not yet been reported. There are more than 120 GAS M serotypes reported thus far (27), which raises a concern about the emergence of serotype replacement, as observed in the use of type-specific pneumococcal vaccines in humans (28). An experimental vaccine targeting the J8 conserved domain of M protein conjugated to diphtheria toxoid has shown protection against heterologous challenge (17).

Several non-M-protein vaccine candidates have been identified and described in the literature (6, 10). Despite showing protection in animal models, with several antigens being widely conserved across serotypes, a non-M-protein vaccine candidate has yet to progress into human clinical trials (11, 29). One challenge in the development of GAS vaccines is the lack of a standardized animal model that can truly mimic human infection (29). This has resulted in the use of various models of disease for assessment of vaccine efficacy, which have been known to deliver conflicting results. Immunogenicity studies with the streptococcal serum opacity factor (SOF) showed the generation of rabbit opsonizing antibodies following immunization with SOF emulsified in complete Freund’s adjuvant, and protection in mice against intraperitoneal lethal challenge (30). However, in a subsequent study, intranasal immunization with SOF adjuvanted with cholera toxin B yielded no protection following intranasal challenge, despite the generation of antigen-specific antibodies (31). Similarly, while intranasal immunization with the fibronectin binding protein I (SfbI) provided protection in a murine intranasal challenge model (31), it failed to provide protection in a murine skin challenge model (32). Identification of GAS antigens that provide protection across different challenge models would support the evaluation of such vaccine candidates in clinical trials.

Here we selected a set of promising antigens that have shown protection in at least one murine model of infection and compared these in two different mouse challenge models (Table 1). ADI and TF are highly conserved proteins localized on the GAS surface that previously showed protection against homologous and heterologous intraperitoneal GAS challenge (7). SLO, SpyCEP, and SCPA are important virulence factors of GAS, which have also been found to be widely conserved across GAS serotypes and have shown protection in various mouse models of infection (8, 33–35). The J8 peptide of M protein, conjugated to diphtheria toxoid has been shown to provide protection in mouse models and to not elicit cross-reactive antibodies against human heart proteins (16, 17, 36). Group A carbohydrate incorporates a polyrhamnose backbone with *N*-acetylglucosamine (GlcNAc) side chains. GAC is found across all GAS serotypes and has been shown to elicit functional antibodies able to confer protection (37). Existing concerns regarding host cross-reactive antibodies that recognize GlcNAc (38, 39) discouraged the use of GAC as a vaccine candidate and prompted the analysis of a GAC variant lacking GlcNAc (ΔGAC) (18). ΔGAC was able to elicit antibodies that showed protection via passive immunization (18).

**TABLE 1:** Vaccine candidates used in this study

Antigen	Function	Inactive form	Reference(s)
ADI	Arginine deiminase.	ADI D277A	7, 12
TF	Ribosome-associated chaperone. Important for protease secretion and activation.		7, 8
SLO	Pore-forming cytolysin. Binds and damages cell membranes, resulting in lysis of the host cell.	SLO P427L W535F	8, 13
SpyCEP	Serine protease cleaves IL-8, interfering with neutrophil recruitment to the site of infection.	SpyCEP D151A S617A	8, 14, 35
SCPA	Subtilisin-like protease. Cleaves C5a, interfering with neutrophil recruitment to the site of infection.	SCPA D130A S512A	8, 15, 33
J8	12-amino-acid peptide from the C-repeat region of M protein.		17, 36, 57, 68
ΔGAC	Group A carbohydrate lacking *N*-acetylglucosamine (GlcNAc) side chain.		18

The experimental vaccines Combo#5, J8-ADI, and ΔGAC-ADI protected BALB/c mice from skin challenge, but they did not induce protection in the *Alb*PLG1 mouse model of invasive disease. Immunization of BALB/c and *Alb*PLG1 mice with M1, Combo#5, J8-ADI, and ΔGAC-ADI elicited significant antigen-specific murine antibodies against each antigen compared to PBS-immunized mice. Such antibodies were also able to bind to the surfaces of GAS and to enhance killing of GAS in the presence of human blood. The presence of antibodies that can promote opsonophagocytosis has been previously correlated with the ability to protect against infection (17, 24) and has been suggested as an immune correlate to be measured in clinical trials (40). Some studies using non-M-protein vaccine candidates have challenged the concept of opsonizing antibodies as being the main correlate of protection (41, 42). In this work, the most effective opsonizing antibody response was generated by M1 protein, which correlated with protection against GAS challenge in the *Alb*PLG1 invasive model. The lower opsonic activity observed for antibodies raised by J8-ADI and ΔGAC-ADI (Fig. 3) correlates with reduced detection of antibody binding to the surfaces of GAS (Fig. 2). On the other hand, a significant albeit lesser opsonizing antibody response was generated by Combo#5 compared to M1, but protection was not apparent in the invasive model of disease. To explain these observations, we speculate that the level of opsonizing antibody generated may be important but perhaps not the only immune effector mechanism necessary to prevent lethal infection. Utilization of different adjuvants that can elicit broader humoral and cellular immune responses may lead to protective efficacy in the invasive disease model. An alternate explanation for the impressive level of protection granted by M1 immunization may be due to the ability of anti-M1 antibodies to block the strong inflammatory response triggered by M protein during infection (43). Blocking of this inflammatory effect could potentially prevent vascular leakage and multiorgan failure, promoting protective efficacy. However, additional studies are required to confirm this hypothesis.

Vaccine efficacy was evaluated using two different murine models of disease. The first model, using BALB/c mice, resembles a superficial non-life-threatening skin infection. This model requires the use of mouse-passaged GAS strains, which have been adapted to the murine host. The second model employed here is the humanized plasminogen murine model. It is well established that activation of human plasminogen by GAS streptokinase is key for systemic dissemination. GAS streptokinase has a greater affinity for human plasminogen than mouse plasminogen; therefore, mice that express human plasminogen are more susceptible to GAS dissemination with nonpassaged GAS strains (44, 45). Each model represents a different disease manifestation, and therefore, the immune mechanisms required for protection may differ for each model. However, the protection afforded by M1 immunization in this study suggests the possibility of developing a vaccine that can confer protection against several clinical manifestations.

Lack of understanding of innate and adaptive immunity following infection in humans has resulted in a lack of standardized correlates of protection (29). An alternative to address this issue is the use of nonhuman primate (NHP) models to characterize immune responses against GAS in an animal model biologically much closer to humans. Experimental GAS colonization in the upper and lower respiratory tracts of NHPs has been reported in rhesus and cynomolgus macaques (46, 47) and in baboons (48). Moreover, NHPs show clinical symptoms of pharyngitis and tonsillitis (46, 47). NHPs may thus represent a powerful tool to investigate immune markers and correlates of protection during streptococcal pharyngitis and ultimately to assess vaccine efficacy (47, 49). During the 1970s, human trials were also carried out where naive and experimentally vaccinated volunteers were infected with GAS (50–52). Controlled infection of volunteers allowed important observations about M-protein immunity and clinical symptoms associated with GAS pharyngeal infection. Following the ban enforced by the U.S. Food and Drug Administration, this type of study has not been undertaken for almost 30 years. Recently, there is increased interest in the development of a GAS human challenge model, with the aim of acquiring better understanding of human immunity to GAS and ultimately to accelerate assessment of vaccine efficacy (53).

Human clinical trials represent huge economic investments. Therefore, a comprehensive portfolio of vaccine antigen efficacy evidence needs to be available. A combination of convincing preclinical data in small-animal models, NHPs, and potentially humans may represent one pathway to progress safe and efficacious GAS vaccine candidates into human clinical trials. We observed a clear difference in the level of protection granted by the experimental vaccine candidates Combo#5, J8-ADI, and ΔGAC-ADI in two mouse models of GAS infection. These results raise important questions regarding the use of mouse models to assess the efficacy of GAS vaccines and the lack of uniformity within the field, particularly as new protective antigens continue to be discovered (54, 55). We strongly believe that correlates of protection in humans and the use of standardized animal models of protection should be openly discussed among investigators and pharmaceutical interests in order to develop the best possible GAS vaccine for safe and efficacious use in humans.

## MATERIALS AND METHODS

### Bacterial strains and growth conditions.

For recombinant protein expression, *Escherichia coli* BL21 Star (DE3) was grown in Luria-Bertani medium (LB) with antibiotic selection as appropriate. *Streptococcus pyogenes* M1T1 5448 strain, an invasive clinical isolate (56), was grown in Todd-Hewitt medium supplemented with 1% (wt/vol) yeast extract (THY). *S. pyogenes* pM1.200, a mouse-passaged M1 streptomycin-resistant reference strain (57) was grown in THY supplemented with 1% (wt/vol) neopeptone and in the presence of streptomycin (200 µg/ml) when required.

### Expression and purification of streptococcal antigens.

SpyCEP (amino acids 40 to 683, D151A S617A) sequence was cloned into the pET151/D-TOPO vector (Invitrogen) following amplification from GAS 5448 genomic DNA and QuikChange site-directed mutagenesis (Agilent Technologies). SCPA (amino acids 40 to 1039, D130A S512A) sequence was cloned into the pET151d vector by sequence- and ligation-independent cloning (SLIC) using synthetic double-stranded DNA (dsDNA) (gBlock; Integrated DNA Technologies) for the 5′ and 3′ sequences and a PCR-amplified internal sequence from SF30 genomic DNA. SLO (amino acids 1 to 571) cloned into pET-15b (58) was modified by site-directed mutagenesis to incorporate P427L and W535A mutations. ADI (amino acids 1 to 411, D277A) was previously cloned into pET151/D-TOPO (12). TF (amino acids 1 to 427) has been cloned into pET151/D-TOPO (7). The gene encoding M1 protein (amino acids 13 to 455) was cloned into pGEX-2T (GE Healthcare Life Sciences), incorporating a carboxy-terminal 6×His tag (9).

ADI, TF, SpyCEP, SLO, and SCPA antigens were expressed in *E. coli* BL21 Star (DE3) cells and purified by immobilized metal ion affinity chromatography (IMAC). Bacterial endotoxins were removed during IMAC by supplementing washing buffers with 0.1% (vol/vol) Triton X-114 (Sigma) (59) or by incubating IMAC-purified proteins with Pierce high-capacity endotoxin removal resin (Thermo Fisher Scientific) following the manufacturer’s protocol. To prepare antigens for ELISA, tobacco etch virus (TEV) protease was used to cleave the His tag from purified ADI, TF, SpyCEP, and SCPA; uncleaved protein and TEV were removed by IMAC. Thrombin protease (Sigma-Aldrich) was used for His tag removal from SLO, followed by size exclusion chromatography and IMAC to remove thrombin and uncleaved SLO, respectively. M1 protein was purified as described previously (9). The final protein concentration was determined using a Direct Detect infrared spectrometer (Millipore). Endotoxin levels were measured using the Pierce *Limulus* amebocyte lysate (LAL) chromogenic endotoxin quantitation kit (Thermo Fisher Scientific).

### Peptide and carbohydrate conjugation to ADI.

J8 peptide was commercially sourced (China Peptides Co.) and conjugated to purified ADI using *N*-(ε-maleimidocaproyloxy)sulfosuccinimide ester (Sulfo-EMCS; Thermo Fisher Scientific) following the manufacturer’s protocol. The ratio of J8 peptide to ADI carrier protein was determined using amino acid analysis (Australian Proteome Analysis Facility) and found to average three peptide molecules per ADI molecule. This ratio translates into a dose of 5 µg of J8 and 25 µg of ADI delivered per vaccinated mouse.

Streptococcal group A carbohydrate lacking *N*-acetylglucosamine (GlcNAc) side chain (ΔGAC) was purified from the GAS 5448Δ*gacI* strain as previously reported (18). Purified ΔGAC was directly conjugated to ADI by cyanylation using 1-cyano-4-dimethylaminopyridinium tetrafluoroborate (CDAP) (Sigma) (60). Briefly, ΔGAC in lipopolysaccharide (LPS)-free water was activated by slowly adding CDAP while vortexing. After 30 s, the pH was raised to pH 8 with triethylamine. At 2.5 min, purified ADI was added, and the reaction mixture was incubated for 4 h at room temperature. The reaction was quenched with excess glycine, and the ΔGAC-ADI conjugate was further purified by size exclusion chromatography. Carbohydrate concentration in the conjugate was measured by the phenol-sulfuric acid method using rhamnose as a standard (61). Specifically, 4.6 µg of ΔGAC was found to be conjugated to 30 µg of ADI, which was the dose delivered per vaccinated mouse.

### Immunization and challenge.

Five groups (*n* = 10 for all groups) of BALB/c mice and transgenic humanized plasminogen mice heterozygous for the human plasminogen gene (*Alb*PLG1) were immunized intramuscularly on days 0, 21, and 28 with 30 µg of total protein adjuvanted with alum (Alhydrogel [2%]; Brenntag) at a 1:1 ratio (50-µl immunization dose)/mouse. The negative-control group received PBS in alum as a sham vaccine. Serum samples were taken before immunization and on day 35. On day 42, immunized mice were challenged with M1 GAS. BALB/c mice were infected cutaneously with 1 × 10^6^ CFU of GAS pM1.200 as previously described (62). At days 3 and 6 postinfection, five mice per group were euthanized to obtain skin, blood, and spleen samples for CFU quantification. Two *Alb*PLG1 mice from the M1 group and one mouse from the Combo#5 group (Combo#5 is the five-component SLO, SpyCEP, SCPA, ADI, and TF vaccine) and the ΔGAC-ADI group were lost prior to challenge (e.g., did not recover from anesthesia) and were excluded from survival analysis. Humanized plasminogen mice (*n* = 10 for the PBS and J8-ADI groups; *n* = 9 for the Combo#5 and ΔGAC-ADI groups; *n* = 8 for the M1 group) were infected subcutaneously with 1.3 × 10^8^ CFU of GAS strain 5448, and survival was monitored for 10 days (63).

### ELISA.

Individual protein antigens (His tags removed) at 5 µg/ml in carbonate coating buffer (50 mM Na_2_CO_3_-NaHCO_3_, pH 9.6), were adsorbed to Titertek polyvinyl chloride (PVC) microplates (M.P. Biomedicals) using 100 µl per well overnight at 4°C. Plates were blocked using 5% (wt/vol) skim milk in phosphate-buffered saline (PBS) containing 0.05% (vol/vol) Tween 20 (90 min, 37°C) and incubated with mouse sera (90 min, 37°C). Antigen-specific mouse antibodies were detected with horseradish peroxidase (HRP)-conjugated goat anti-mouse IgG antibody (Thermo Fisher Scientific) and SIGMA*FAST o*-phenylenediamine dihydrochloride (OPD) (Sigma-Aldrich) as an HRP substrate with absorbance measured at 450 nm. Endpoint titers were determined as the highest dilution of serum for which the absorbance was 3 standard deviations above the mean optical density of blank wells.

Purified ΔGAC was activated with 15 mM NaIO_4_ in sodium acetate buffer (0.1 M sodium acetate [pH 5.5]) for 30 min at room temperature. The reaction was stopped with ethylene glycol, and activated ΔGAC was dialyzed against sodium acetate buffer. Costar carbohydrate binding plates (Corning) were coated with 100 µl of activated GAC (10 µg/ml) in sodium acetate buffer for 1 h at room temperature. Plates were blocked with 1% (wt/vol) bovine serum albumin (BSA) in 50 mM Tris (pH 8.2) for 30 min at room temperature. Mouse serum samples in PBS supplemented with 10% (vol/vol) goat serum were added and incubated at 37°C for 90 min. Detection of ΔGAC-specific mouse antibodies and determination of endpoint titers was done as described above.

### Detection of antibody binding to the GAS surface by flow cytometry.

GAS strains were grown to mid-logarithmic phase (optical density at 600 nm [OD_600_] of 0.6), washed in PBS, and blocked using nonspecific human IgG (200 μg/ml; Merck Millipore) in PBS with 3% (wt/wt) BSA (3% BSA/PBS) (1 h, 4°C). Bacterial cells were washed and resuspended in PBS. A volume of 0.3 ml of bacterial suspension in PBS (OD_600_ of 0.6) was incubated overnight at 4°C in 100 µl of pooled mouse sera and then diluted 1:50 in 0.3% BSA/PBS (wt/vol). Pellets were washed in PBS and resuspended in 100 µl of a 1:200 dilution of goat anti-mouse IgG (H+L) conjugated to Alexa Fluor 488 (Thermo Fisher Scientific) in 0.3% BSA/PBS (wt/vol). Cells were washed with PBS and fixed in 1.5% paraformaldehyde/PBS (wt/vol). A total of 50,000 events were analyzed using a BD Accuri C6 flow cytometer (BD Biosciences), and further analysis was undertaken using FlowJo software (Tree Star Inc.).

### Bacterial opsonization assay.

The indirect bactericidal activity of mouse antibodies was measured as previously described with minor changes (9). Briefly, GAS strains were grown until early logarithmic phase (OD_600_ of 0.4). Pooled heat-inactivated serum from immunized BALB/c mice was incubated for 20 min at room temperature with GAS pM1.200 diluted in PBS to a 1 × 10^−4^ dilution. Likewise, pooled heat-inactivated serum from immunized humanized plasminogen *Alb*PLG1 mice was incubated with GAS 5448 diluted in PBS to a 5 × 10^−5^ dilution. Fresh human blood from a volunteer, previously tested to support the growth of both strains, was added, and samples were incubated (3 h, 37°C) with end-to-end rotation before being plated out in triplicate for CFU determination. Opsonic activity of the immune sera (percent reduction in mean CFU) was calculated as follows: (1 − CFU in the presence of vaccine immune sera/mean CFU in the presence of sham-immunized mouse sera) × 100. Three independent replicates were performed for each treatment.

### Statistical analysis.

Differences in antigen-specific endpoint titers were analyzed using the two-tailed Mann-Whitney U test with *P* of <0.05 considered statistically significant (GraphPad Prism 6). Flow cytometry data were analyzed using the probability binning algorithm in FlowJo 10.1 (Tree Star Inc.), a cutoff value of *T*(*X*) of 100 was empirically determined, and samples having *T*(*X*) of >100 were considered significant (*P* < 0.01) (99% confidence) (64, 65). [*T*(*X*) is a statistic metric developed to provide an indication of the probability that two populations differ from each other by using the probability binning algorithm. The higher the value of *T*(*X*), the less alike the populations are.] Survival times in the indirect bactericidal assay were compared using one-way analysis of variance (ANOVA) corrected for multiple comparisons using Dunnett’s test, with *P* < 0.05 considered statistically significant (GraphPad Prism 6). Differences in bacterial persistence and dissemination were analyzed using the Kruskal-Wallis test corrected for multiple comparisons using Dunn’s test (GraphPad Prism 6). Murine survival curves were analyzed using the Mantel-Cox log rank test with *P* < 0.05 considered statistically significant (GraphPad Prism 6).

### Ethics approvals.

All animal procedures were conducted according to the *Australian Code for the Care and Use of Animals for Scientific Purposes* (66). Procedures using BALB/c mice were approved by the Griffith University Animal Ethics Committee, and procedures using humanized plasminogen *Alb*PLG1 mice were approved by the University of Queensland Animal Ethics Committee. Human blood donation for use in indirect bactericidal assays was conducted in accordance with the *National Statement on Ethical Conduct in Human Research* (67), complied with the regulations governing experimentation on humans, and was approved by the University of Queensland Medical Research Ethics Committee.
